# Observations on the Topography, Climate, and Prevalent Diseases of the Island of Jersey; the Result of Meteorological Observations and General Practice during Thirteen Years

**Published:** 1837-10

**Authors:** 


Art. IV.—*Observations on the Topography, Climate, and prevalent Diseases of the Island of Jersey; the Result of Meteorological Observations and general Practice during thirteen Years.* By G. S. Hooper, m.d.—*London,* 1837. 8vo. pp. 199.

This is a production very creditable to the author, and will be very useful to those among the large body of our countrymen that annually visit Jersey, who, before proceeding to take up their abode in that island, deem the nature of the climate an important consideration. On this subject Dr. Hooper's book contains full information; and, although the author will not entirely escape the charge of partiality in favour of his own place of residence, yet we must admit that his statements are, on the whole, given with candour. Notwithstanding the general commendations of the climate scattered throughout the work, we think the consideration of its details will leave an impression on the mind of his medical readers unfavorable to Jersey as a residence for invalids. Although, like the Land's End, (whose climate it greatly resembles,) it presents great equability of temperature, diurnal, monthly, and annual; and, although this is extremely mild, both in summer and winter, yet, as in the Land's End, the other elements of climate are of a decidedly unfavorable character. Owing to its comparative flatness and nakedness, it lies exposed to every wind; while the frequency of its rains, and its geographical and topographical relations, render it extremely damp. The account given of the diseases by Dr. Hooper corroborates, in a striking manner, this unfavorable view of the climate of Jersey; those diseases which are most certainly traceable to a raw and uncertain climate being particularly prevalent here,—viz. catarrh, bronchitis, and rheumatism.

"For this period of the year" (winter and spring,) "is extremely variable; one day wearing, as if by anticipation, the cheering attractions of a summer sky; whilst the next, cold, dark, and rainy, turns the disappointed mind back to the irksomeness of an inclement season. By reason of these variations, . . . the fashion of low dresses, short sleeves, and bare legs, for children, is admissible, on no sound principles, in this island." (P. 153.) . . . "Among the diseases incidental to infancy and childhood, I have allotted the first rank, in the order of comparative prevalence, to catarrhal affections and intestinal irritation." (P. 147.) . . . . "That acute inflammation of the air-passages is a prominent disease among the children in this island, cannot admit of a doubt." (P. 149.) . . "Concerning bronchitis" [in adult age,] "I have nothing to state beyond the fact of its great prevalence." (P. 172.) . . . "Rheumatism is so general in these parts that it might, with some reason, be viewed in the light of an endemical complaint. . . In the male sex, excepting hernia, no single disease constitutes so frequent a cause of disability for the military service. . . The exemptions owing to this cause may, without exaggeration, be estimated at one-tenth of the whole number of those which are final." (P. 173.)

By way of making amends for this unfavorable view of diseases universally admitted to be of climatorial origin, the author endeavours to show that scrofula is not more prevalent in the island than elsewhere, as, it seems, "has been asserted, and very generally credited;" and that consumption is rather less prevalent than elsewhere. Neither of these positions, however, is satisfactorily made out; and, from what we ourselves know of the great prevalence of these diseases in climates almost identical with that of Jersey, we shall require evidence of a very different kind from that adduced before we can admit the legitimacy of the author's inferences.

A striking defect in this work as a topographical document, is the total absence of statistical details respecting the births and deaths of the population. In so small a community, these documents, one would imagine, might have been easily obtained; and the results, owing to the isolation of the inhabitants, would have been highly interesting and important.

